# Characterization of the *Trans* Watson-Crick GU Base Pair Located in the Catalytic Core of the Antigenomic HDV Ribozyme

**DOI:** 10.1371/journal.pone.0040309

**Published:** 2012-06-29

**Authors:** Dominique Lévesque, Cédric Reymond, Jean-Pierre Perreault

**Affiliations:** Département de Biochimie, Faculté de Médecine et des Sciences de la Santé, Université de Sherbrooke, Sherbrooke, Québec, Canada; Beckman Research Institute of the City of Hope, United States of America

## Abstract

The HDV ribozyme’s folding pathway is, by far, the most complex folding pathway elucidated to date for a small ribozyme. It includes 6 different steps that have been shown to occur before the chemical cleavage. It is likely that other steps remain to be discovered. One of the most critical of these unknown steps is the formation of the *trans* Watson-Crick GU base pair within loop III. The U_23_ and G_28_ nucleotides that form this base pair are perfectly conserved in all natural variants of the HDV ribozyme, and therefore are considered as being part of the *signature* of HDV-like ribozymes. Both the formation and the transformation of this base pair have been studied mainly by crystal structure and by molecular dynamic simulations. In order to obtain physical support for the formation of this base pair in solution, a set of experiments, including direct mutagenesis, the site-specific substitution of chemical groups, kinetic studies, chemical probing and magnesium-induced cleavage, were performed with the specific goal of characterizing this *trans* Watson-Crick GU base pair in an antigenomic HDV ribozyme. Both U_23_ and G_28_ can be substituted for nucleotides that likely preserve some of the H-bond interactions present before and after the cleavage step. The formation of the more stable *trans* Watson-Crick base pair is shown to be a post-cleavage event, while a possibly weaker *trans* Watson-Crick/Hoogsteen interaction seems to form before the cleavage step. The formation of this unusually stable post-cleavage base pair may act as a driving force on the chemical cleavage by favouring the formation of a more stable ground state of the product-ribozyme complex. To our knowledge, this represents the first demonstration of a potential stabilising role of a post-cleavage conformational switch event in a ribozyme-catalyzed reaction.

## Introduction

**Figure 1:**
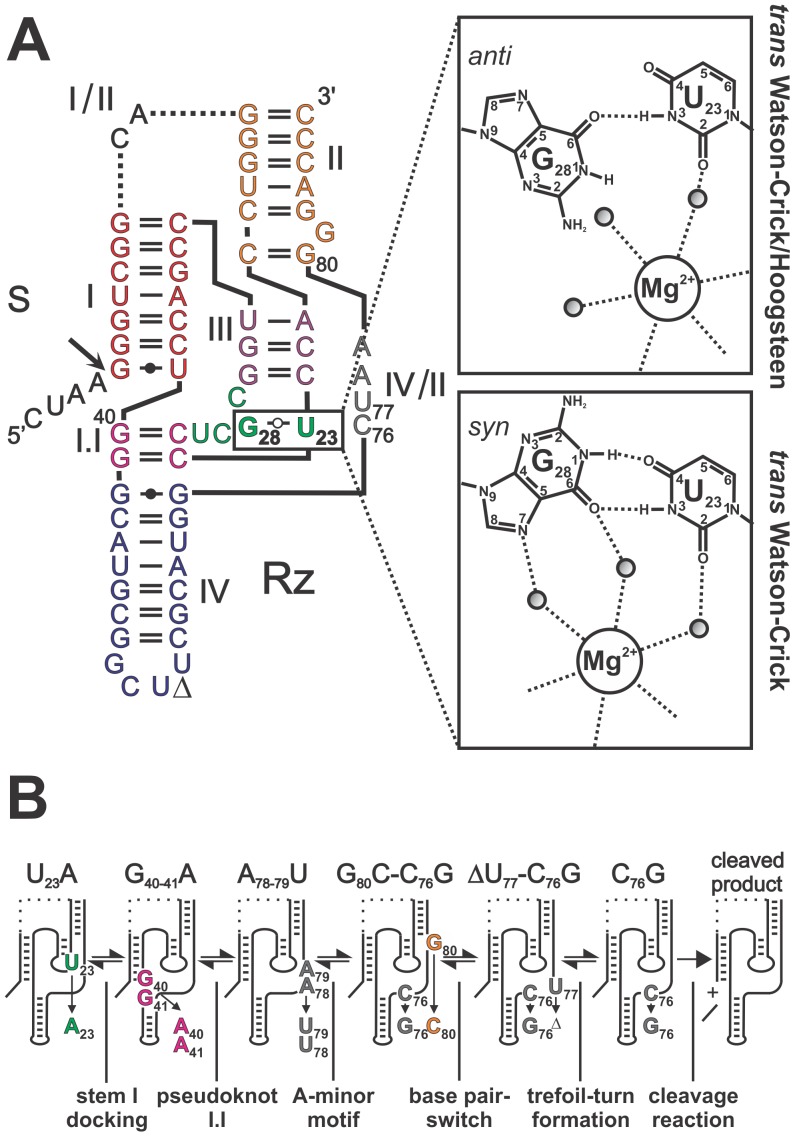
Structure and folding pathway of the antigenomic HDV Rz. (**A**) Nucleotide sequence and secondary structure of the HDV Rz antigenomic version used in this study. The ribozyme and the substrate are denoted by Rz and S, respectively. The cleavage site is indicated by the arrow. The harmonized nomenclature using roman numerals and colors for the stems is indicated [2]. The dotted line represents the junction I/II that was removed to generate a *trans*-acting version. The inset shows the H-bonds involved in the formation of either the *trans* Watson-Crick/Hoogsteen, in *anti* conformation, or the *trans* Watson-Crick, in *syn* conformation, GU base pair between U_23_ and G_28_ of loop III. The atom numbering for both nucleotide bases is shown. A putative magnesium binding site [21] is also shown. (**B**) Schematic representation of the secondary structure of the mutants capable of halting the folding pathway of HDV Rz at several stable intermediates. The mutated nucleotides are indicated.

Understanding the RNA structure/function relationship is critically important for further development in the fields of both molecular and cellular biology. Among the processes underlying RNA function, RNA folding is the most essential and the most challenging. In addition, the study of RNA folding is paramount to improving our knowledge and understanding of RNA related-diseases, which in turn will have a major impact on human health. Ribozymes (Rz) are both interesting and convenient molecules with which to study the RNA structure/function relationship because even the slightest modification in a ribozyme’s structure generally affects its catalytic properties. Among ribozymes, the hepatitis delta virus (HDV) Rz has perhaps been the most extensively studied [1]. Conversion of the HDV self-cleaving strand from a *cis*-acting to a *trans*-acting system by separating the Rz and substrate (S) domains significantly facilitated its study (Figure 1A). Both structure/function assays and structural studies have helped to elucidate its double-pseudoknot secondary structure, a structure that is composed of two stems (stems I and II, the latter forming a pseudoknot in the *cis*-acting version), two stem-loops (III and IV) and three single-stranded junctions (I/II, I/IV, and IV/II) (Figure 1A) [2]. Both junction I/IV and loop III are single-stranded in the initial stages of folding, but subsequently become involved in the formation of the pseudoknot I.I [3].

The generation of a specific set of mutants capable of halting the folding pathway at several stable intermediates has enabled further characterization of the folding pathway (Figure 1B). Briefly, recognition of the S by the Rz leads to the formation of stem I. Five sequential conformational steps then produce the catalytically active structure. The first of these steps is the docking of stem I within the catalytic core that brings together the middle section of stem I with both the C_22_ and U_23_ residues of loop III [4]. The second is the formation of pseudoknot I.I, a key step for helix packing [5]. The third is the formation of the A-minor motif between two adenosines of junction IV/II and the minor groove of stem III [6]. Finally, the fourth and fifth steps are, respectively, the formation of a base pair-switch (bp-switch), which relaxes the phosphodiester backbone of junction IV/II and positions the catalytically active C_76_ deep inside the catalytic core, and the trefoil-turn pivoting around the extruded U_77_ [7]–[9]. The bp-switch, which occurs only in the antigenomic version, substitutes the C_19_-G_81_ bp for a new C_19_-G_80_ bp at the bottom of stem II. Only then does the catalytically active C_76_ enter into action. Kinetic traps have been shown to occur along this folding pathway; however, any molecules caught in a kinetic trap can be reintegrated into the folding pathway [4].

Divalent metal ions have been shown to be essential for proper HDV Rz catalysis at physiological pH [10]. Several studies have provided indications of the rough localization of the Mg^2+^ cations, albeit without revealing their precise positions. Specifically, one is located between stems I and III, another moves along the IV/II junction and a third interacts with the GU Wobble bp located adjacent to the scissile bond [11].

Even if the description of the folding pathway of the antigenomic HDV Rz is, by far, the most complex folding pathway elucidated to date for a small ribozyme, it is likely that other essential steps remain to be discovered. One of the critical steps that remains to be elucidated is the formation of the unusual *trans* Watson-Crick (*t*WW) (also known as reverse Wobble) GU base pair found in loop III. In the antigenomic HDV Rz, this base pair is produced by the formation of two H-bonds between the O6 and N1H of G_28_ with the N3H and O4 of U_23_, respectively (Figure 1A, inset) [12]. This *t*WW GU base pair is highly conserved in both the genomic and antigenomic Rz [1], in the HDV-like sequences found within both the mammalian cytoplasmic polyadenylation element binding protein 3 (CPEB3) and the CPEB3-like genes [13], [14] as well as in the HDV-like sequences found in both the R2 and L1Tc retrotransposon families [15], [16]. The absolute requirement for these two nucleotides is illustrated by the fact that a bioinformatic search for putative HDV-like motifs in all kingdoms of life was based on the presence of the six invariant nucleotides found in both HDV and human CPEB3 Rz, and that the aforementioned two residues were included among the 6 invariant ones [17]. Moreover, an unbiased *in vitro* selection experiment in which 25 nucleotides, including all of the positions of loop III of the antigenomic HDV Rz, were randomized provided additional evidence of the importance of both the U_23_ and G_28_ residues as they were almost perfectly conserved among the 330 clones sequenced [18]. Taken together, these data demonstrate the absolute requirement for the presence of these two nucleotides within loop III for the integrity of HDV Rz.

Using a cleaved-product RNA, an NMR experiment based on a genomic/antigenomic chimeric HDV Rz indicated that U_20_ and G_25_ (which correspond to U_23_ and G_28_ in the antigenomic version) of loop III form a *t*WW GU base pair [19]. On the other hand, crystallographic data from a cleaved-product RNA derived from genomic HDV Rz revealed that U_20_ and G_25_ face each other in the *t*WW orientation without forming any H-bonds (see PDB 1DRZ) [6]. Moreover, in this crystal, G_25_ adopts the more compact and unusual full *syn* conformation. The formation of this *t*WW base pair was not observed using a pre-cleaved complex that was created by replacing C_75_ (i.e corresponding to C_76_ of the HDV antigenomic Rz) with an uridine, but instead the formation of a *trans* Watson-Crick/Hoogsteen (*t*WH) base pair (by forming an H-bond between the O6 of G_25_ with the N3H of U_20_) with the G in the more usual *anti* configuration was detected (see PDB 1SJ3 and Figure 1A inset) [7]. Consequently, it was proposed that this *t*WW GU base pair is likely formed after the chemical step. However, crystal analysis of a *trans*-acting genomic/antigenomic chimeric ribozyme in which only U_−1_ of the substrate was replaced by a deoxyuridine (which results in the production of an uncleavable substrate) demonstrated that U_20_ and G_25_ interact together prior to cleavage in a *trans* Watson-Crick configuration with the G in the *syn* configuration (see PDB 3NKB and Figure 1A inset) [20]. Therefore, it seems that these bases can pair by forming either a *trans* Watson-Crick/Hoogsteen base pair with the G in *anti* conformation, or a *trans* Watson-Crick base pair with the G in *syn* conformation. Moreover, it was shown that this base pair likely acts as a magnesium binding pocket in which the divalent ion neutralizes the negative charges near the *t*WW GU, thereby helping to shift the pK_a_ of the catalytic C_75_ [21].

Finally, molecular dynamic (MD) simulations have led to suggestions that this magnesium is likely to be chelated, and that it contributes to the catalysis [22]. It is noteworthy that all of these results are derived from either crystal/NMR analyses, or MD simulation, while no direct evidence about the *t*WW GU base pair formation has been demonstrated in solution. In order to clarify the discrepancy concerning exactly when the *t*WW GU base pair of loop III forms, as well as to confirm, in solution, its crucial role in the HDV Rz catalysis, several experiments directed towards characterizing its formation within the HDV antigenomic Rz were performed.

## Results

### Kinetic Analysis of All Possible *Trans* Watson-Crick GU Mutants

**Figure 2:**
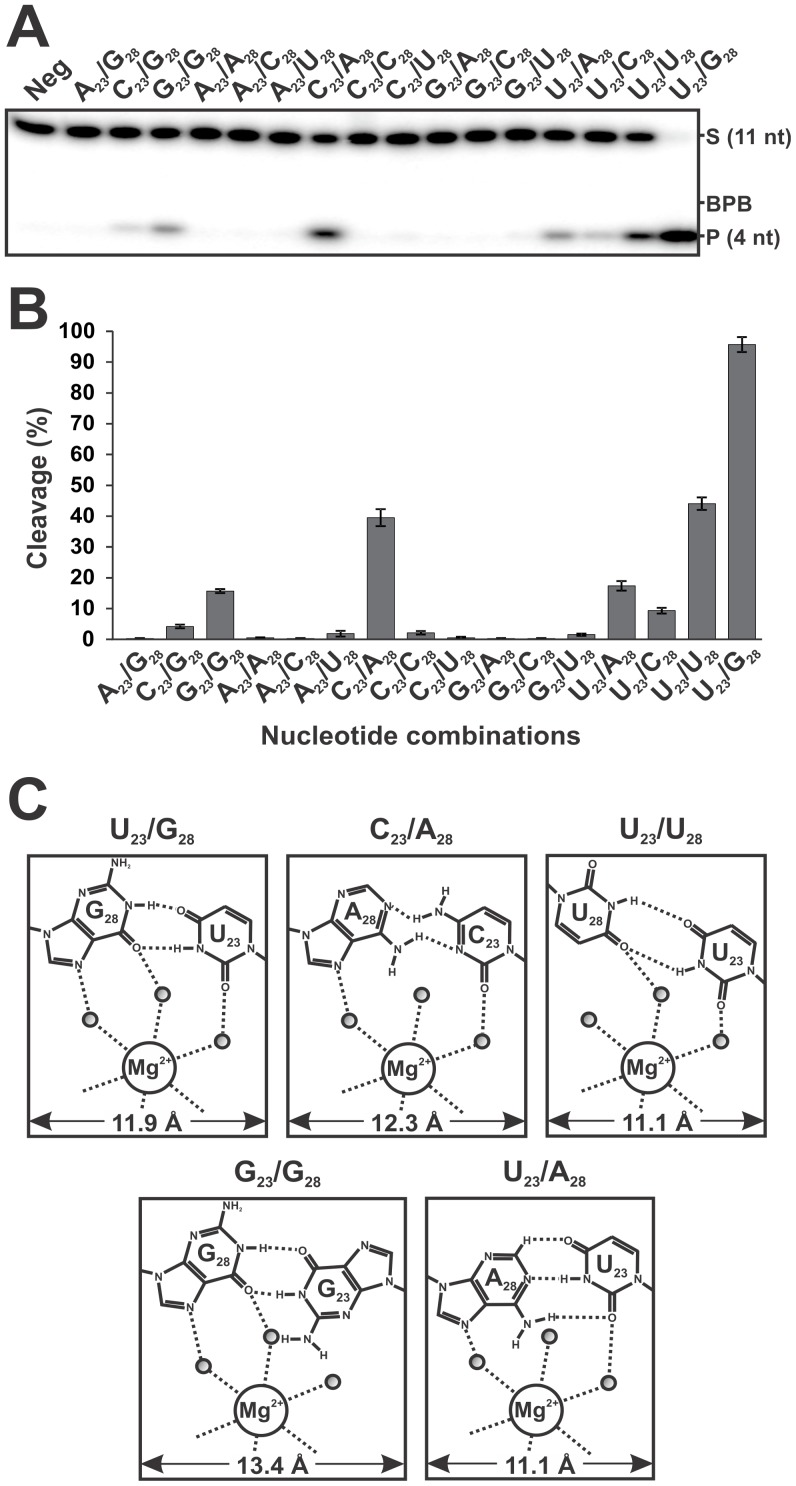
Cleavage activities of all of the ribozymes mutated in positions 23 and 28. (**A**) Autoradiogram of the denaturing PAGE gel used for the analysis of the cleavage reactions. In all cases, the ribozymes (100 nM) were incubated for 2 h with trace amounts of 5′-end-labeled substrate (<1 nM). The positions of the bromophenol blue dye (BPB), the 11-nt substrate (S) and the 4-nt product (P) are indicated. “Neg” represents a cleavage reaction performed in the absence of any Rz. (**B**) Graphical representation of the cleavage percentages for all of the 16 nucleotide combinations examined. The values are means of at least 2 different experiments and the error bars represent the standard deviation (**C**) Putative H-bond representation and magnesium ion binding site of the wild-type (U_23_/G_28_) and four other nucleotide combinations.

All 508 natural HDV versions of both the genomic and antigenomic polarities harbour U_23_ and G_28_ residues in loop III [23]. In addition, several structure/function studies, including both the deletion and the mutation of these nucleotides, have shown that they are crucial to the ribozyme’s catalytic activity (e.g., [24]–[26]). However, to our knowledge, there is no report of a mutational study in which both residues were simultaneously mutated. In order to investigate whether an alternative base composition is possible for these two positions, mutated ribozymes encoding all 16 possible base/base combinations were synthesized. A strategy based on the production of 16 different PCR-filled DNA oligonucleotide templates, each containing the T7 RNA polymerase promoter and specific, unique nucleotides in positions 23 and 28, and their subsequent use for the transcription of specific *trans*-acting ribozymes, was employed. After production, these ribozymes were used in cleavage reactions under single-turnover conditions that included trace amounts of a 5′-end ^32^P-labeled 11-nt substrate (S) and 100 nM of ribozyme ([Rz]>>[S]). The reactions were incubated for 2 h at 37°C in the presence of 10 mM MgCl_2_, and the reaction products analysed by denaturing polyacrylamide gel electrophoreses (PAGE) (see Figure 2A). While the original ribozyme that included U_23_ and G_28_ exhibited a cleavage level of near 95%, an isosteric reverse Wobble mutant containing the inverted nucleotides (i.e. G_23_ and U_28_) was found to be inactive (<2% cleavage activity). This result demonstrates that both the nucleotide identity and the spatial environment (e.g. a *trans* Watson-Crick or *trans* Watson-Crick/Hoogsteen possible interaction) are more important than the simple retention of an isosteric reverse Wobble base pair. Four of the mutated ribozymes exhibited cleavage activities greater than 15% (Figure 2A,B). Specifically, the mutants including G_23_/G_28_, C_23_/A_28_, U_23_/A_28_ and U_23_/U_28_ exhibited cleavage levels that reached, after 2 h, 16%, 40%, 17% and 44% respectively, and maximum end-point cleavage levels estimated to be 53%, 84%, 75% and 65%, respectively (data not shown). These results indicate that these four mutants retain some cleavage activities, although a longer incubation times can be required in order to reach a significant end-point as compared to that of the wild-type Rz. Among this group of active ribozymes, C_23_/A_28_ is the only double mutant. The combination C_23_/A_28_ has the ability to form a reverse Wobble base pair, as is the case for U_23_/G_28_ [27]. However, the isosteric combination A_23_/C_28_, as is the case of G_23_/U_28_, was found to be inactive, supporting the notion of the importance of both the nucleotide identity and the spatial environment. Importantly, all of the active ribozymes possess base pairs that can form at least two H-bonds in the *t*WW configuration (Figure 2C). The distances between the C1 of the ribose residues of both nucleotides vary only slightly, between 11.1 Å and 13.4 Å, and are similar to the original *t*WW U_23_/G_28_ base pair’s distance of 11.9 Å. Moreover, there is without doubt a notion of spatial orientation that has to be adequate for the proper positioning of the chemical groups involved in the binding and coordination of a Mg^2+^ cation. These particular requirements could also explain the different activities observed for these mutants. Of the eleven other combinations that were found to be inactive, five can be explained by either inadequate distances or spatial environments. Amongst the remaining six combinations, two are base combinations that do not belong to the *trans* Watson-Crick family, and four involve the inactive U_23_A mutant (data not shown). Importantly, these results demonstrate that it is possible to modify the identity of the residues located in positions 23 and 28, although these modifications strikingly affect the cleavage level. It is noteworthy that a *t*WH U_23_/G_28_ base pair, in which the G was found to be in an *anti* conformation, was retrieved in the pre-cleaved genomic HDV Rz crystal [7]. In that case, it could be possible that active base combinations must also be able to adopt this specific base pairing interaction. Of the sixteen base combinations, six are ones that do not belong to the *trans* Watson-Crick/Hoogsteen family and two involve the inactive mutant U_23_A. Amongst the eight other possibilities, five are the active base combinations. The remaining three other left could be removed by the first criteria concerning the *t*WW base pair. Taken together, the active combinations of nucleotides likely must have the possibility of interacting together either in a *trans* Watson-Crick/Hoogsteen or a *trans* Watson-Crick interaction manner.

In order to further characterize the importance of the bases located at both positions, pseudo-first order kinetic analyses of the five active ribozymes (i.e. the original ribozyme and the four mutants) were performed. The k_2_ and K_M’_ values are compiled in Table 1. The wild-type ribozyme that includes the combination U_23_/G_28_ had a k_2_ value of 1.74 min^−1^, which is quite different from the 0.19 min^−1^ previously reported [28]. This difference could be explained by the use of a substrate containing the optimal −4 to −1 nt sequence (i.e. 5′-C_−4_UAA_−1_) [29] which was not the case in the previous work (i.e. 5′-G_−4_GGC_−1_) [28]. The K_M’_ value obtained with this optimal substrate (38.1 nM) also differed significantly from that observed previously (9.1 nM) [28]. When the k_2_ value of the wild-type ribozyme (i.e.U_23_/G_28_) was compared with those of the ribozymes mutated at positions 23 and 28, striking differences, ranging from 232- to 528-fold, were observed (see Table 1). These differences may explain why these mutants have not been found in nature. Conversely, the K_M’_ values were virtually identical, varying only by approximately +/−3-fold (see Table 1). Clearly, an important variation of the k_2_ values is the main explanation for the striking decreases in the catalytic activities of these mutants.

**Table 1:** Kinetic parameters of the active mutant ribozymes.

Rz	k_2_ (min^−1^)	K_M’_ (nM)	k_2_ folddecrease	K_Mg_ (mM)
**U_23_G_28_**	1.74±0.05	38.1±4.0	1	4.4±1.0
**C_23_A_28_**	0.0075±0.0003	21.0±4.5	232	6.1±1.0
**U_23_U_28_**	0.0060±0.0004	10.9±4.2	290	5.9±0.7
**G_23_G_28_**	0.0037±0.0010	46.5±7.3	470	10.0±1.2
**U_23_A_28_**	0.0033±0.0004	115.8±36.2	528	12.3±2.2
**RD U_23_G_28_**	0.36±0.01	79.0±4.3	1*	6.4±1.4
**RD U_23_I_28_**	0.24±0.01	70.4±4.4	1.5*	7.4±1.7
**RD U_23_2AP_28_**	0.018±0.002	44.2±16.4	20*	15.1±1.6
**RD U_23_N7D_28_**	0.0062±0.0005	125.6±34.1	58*	13.8±2.8

* Indicates that all of the RD mixed ribozymes were compared to each other and not with the all RNA Rz.

Finally, it has been suggested that this *t*WW GU base pair could play a central role in the coordination of a magnesium ion, properly positioning it and leading to the catalysis [22]. In order to verify whether or not the nucleotides located in positions 23 and 28 have an impact on the binding of this metal ion, the K_Mg_ values were determined for each of the active mutated ribozymes, and were compared to that of the original ribozyme with the U_23_/G_28_ nucleotide combination (Table 1). This latter ribozyme has an estimated K_Mg_ value of 4.4 mM, which is virtually identical to those of the mutants with the U_23_/U_28_ and C_23_/A_28_ nucleotide combinations (i.e. 5.9 mM and 6.1 mM, respectively; see Table 1). This suggests that these three ribozymes bind Mg^2+^ with equivalent affinities. Conversely, the mutants with the nucleotide combinations G_23_/G_28_ and U_23_/A_28_ exhibited cleavage activities with K_Mg_ values that were increased by an average of 2.5-fold (i.e. 10.0 mM and 12.3 mM, respectively; see Table 1). This is not a highly significant difference; however, it may be indicative of a different Mg^2+^-dependency.

In summary, these results showed that the base pair of loop III can be mutated, but that the substituted bases should have the ability to interact and to form the necessary *t*WH or *t*WW base pair. The best combination, by far in terms of cleavage activity, is the original GU base pair. In addition, varying the base composition at these positions likely influence the binding of either the magnesium interacting with some of the functional groups of the U_23_/G_28_ base pair, or the functionally required Mg^2+^ affinity of a different magnesium binding site.

### Characterization of the Functional Groups within Either the *Trans* Watson-Crick or the *Trans* Watson-Crick/Hoogsteen GU Base Pair

**Figure 3:**
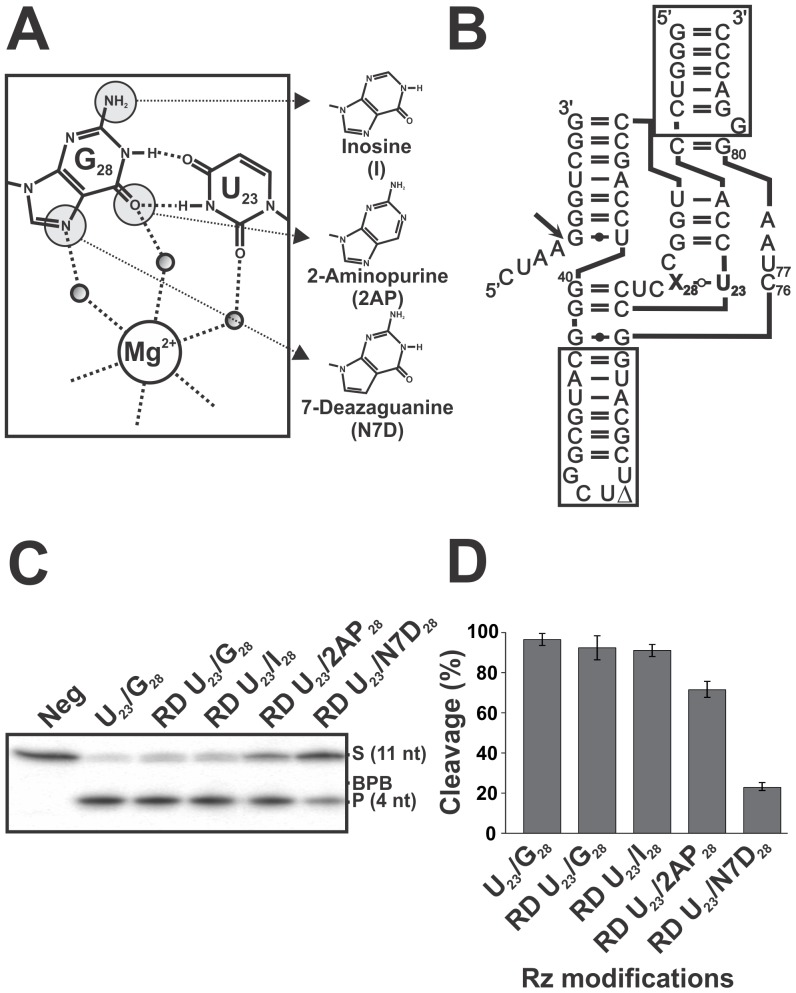
Cleavage activities of the various modified chemical groups of G_28_. (**A**) Schematic representation of the *trans*-Watson-Crick GU base pair (inset) and of three potential substitutions for G_28_. (**B**) Sequence and secondary structure of the original RD mixed ribozyme. The boxed regions represent the DNA parts of the oligonucleotide. (**C**) Autoradiogram of the denaturing PAGE gel used for the analysis of the cleavage reactions of the RD mixed oligonucleotides containing the modified chemical groups. In each case, the ribozyme (100 nM) was incubated for 2 h with trace amounts of 5′-end-labeled substrate (<1 nM) and the reactions were analyzed on denaturing 20% PAGE gels. Neg represents a cleavage reaction performed in the absence of any Rz. The positions of the bromophenol blue dye (BPB), the 11-nt substrate (S) and the 4-nt product (P) are indicated. (**D**) Graphical representation of the cleavage percentages for the reactions shown in (**C**). The values are means of at least 2 different experiments and the error bars represent the standard deviation.

The formation of the two H-bonds of a *t*WW GU base pair involves four different functional groups (Figure 3A). Specifically, the O6 and N1H groups of G_28_ interact with the N3H and O4 groups of U_23_, respectively. It is noteworthy that in the genomic Rz a *t*WH base pair, formed by an H-bond between the O6 of G_25_ with the N3H of U_20_, was found in the pre-cleaved genomic Rz [7]. Furthermore, in the case of the *t*WW GU base pair in loop III, it has been suggested that the putative hydrated Mg^2+^ ion binding is likely stabilized by H-bond interactions with both the N7 and O6 groups of the Hoogsteen edge of the G_28_ residue, as well as with the O2 group of U_23_ [21]. In order to learn more about the involvement of some of these functional groups, mixed RNA-DNA (RD) oligonucleotides, in which different chemical groups were absent, were synthesized. This RD mixed oligonucleotide strategy was previously used for the study of the important 2′-hydroxyl groups (2′-OH) of the antigenomic HDV Rz [28]. Basically, the strategy involves the replacement of the ribonucleotide residues of both stems II and IV by deoxyribonucleotides, thereby reducing the synthesis costs without significantly altering the kinetic constants of the ribozyme (see Figure 3B). To our knowledge, no phosphoramidite possessing chemical substitutions on the uridine located in position 23 that can be of use in this study is commercially available. Consequently, our efforts were concentrated on the chemistry available for the guanosine located in position 28 (see Figure 3A). More specifically, three chemically different species were used: i. inosine (I), in which the NH_2_ group linked to the C2 of guanine is removed; ii. 2-aminopurine (2AP), in which the O6 group of the guanine, which appears to be acceptor of an H-bond involved in the formation of the *trans*-Watson-Crick base pair, is removed; and, iii. 7-deazaguanine (N7D), in which the N7 of guanine is replaced by a carbon residue. This latter modification does not affect the base-pairing between U_23_ and G_28_, but rather the binding of the putative nested Mg^2+^ ion.

The RD mixed oligonucleotides were synthesized, deprotected and purified. The cleavage assays were then performed under single-turnover conditions. The all RNA and the RD mixed ribozymes containing the original U_23_/G_28_ nucleotides were considered as being controls. Both of these ribozymes almost completely cleaved all of the substrate after 2 h of incubation (i.e. >95%) (Figure 3C, D). The three other RD mixed ribozymes all exhibited cleavage activity, although at different levels (Figure 3C, D). The one containing the I_28_ was as active as the original Rz containing the G_28_, reaching a cleavage end-point of 96%. This is in agreement with the idea that the NH_2_ group of G_28_ is not essential to the catalytic action of the ribozyme (see Figure 3A). The RD mixed ribozymes containing the 2AP_28_ and the N7D_28_ substitutions had cleavage end-points that were reduced to 72% and 22%, respectively. These results suggest contributions of both the O6 and N7 groups to the cleavage activity. Pseudo-first order kinetic analyses, performed so as to clarify the contributions of these groups, revealed that the RD mixed ribozyme with the original U_23_/G_28_ base pair cleaved with k_2_ and K_M’_ values of 0.36 min^−1^ and 79 nM, respectively (Table 1). The corresponding values estimated for the RD mixed ribozyme that included an I_28_ residue were almost identical (i.e. 0.24 min^−1^ and 70.4 nM, respectively), supporting the idea that the deleted NH_2_ group is not involved in the interaction that leads to the formation of either the *t*WW or *t*WH GU base pairs. Moreover, no effect in term of K_Mg_ was observed for this substituted RD mixed ribozyme (i.e. its K_Mg_ was 7.4 mM as compared to 6.4 mM for the original RD mixed ribozyme). In the case of the RD mixed ribozyme that included the 2AP_28_ residue, the k_2_ value was 20-fold smaller, while the K_M’_ was only reduced 2-fold as compared to original sequence’s values (i.e. 0.018 min^−1^ and 44.2 nM, respectively). Finally, the RD mixed ribozyme harbouring the N7D_28_ residue demonstrated a 58-fold decrease in k_2_ and a 1.6-fold increase in K_M’_ (0.0062 min^−1^ and 125.6 nM, respectively). The RD mixed ribozyme that included the 2AP_28_ residue possessed a K_Mg_ value of 15.1 mM, while that of the one containing the N7D_28_ residue was 13.8 mM. The observed changes in K_Mg_ is rather mild, indicating that either the O6 and N7 groups of G_28_ are likely involved in coordinating the Mg^2+^ ion inside loop III or that these substitutions induce structural changes in the ribozyme rather than the loss of metal-binding ligand. However, it was surprising to observe that the deletion of the O6 did not have a more pronounced effect based on the H-bond removal in the base pair, particularly for the possible *t*WH base pair where it should theoretically remove the sole H-bond formed (see Figure 1A inset). The absence of a larger effect for the deletion of the O6 group may be an indication that both the formation of the H-bond between the N3H group of U_23_ and the O6 group of G_28_, and that formed by the solvated magnesium ion, could be intimately associated.

### Formation of the *Trans* Watson-Crick GU Base Pair

**Figure 4:**
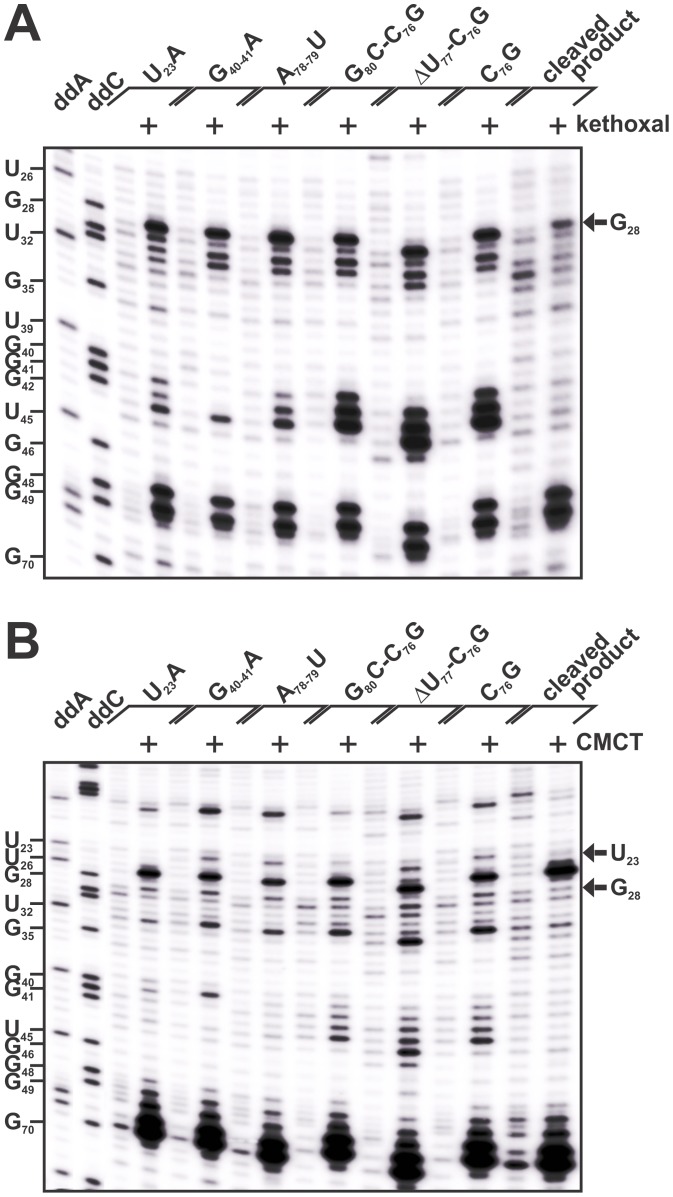
Chemical probing of the *trans* Watson-Crick GU base pair. Various *cis*-acting mutants were folded in the presence of MgCl_2_ and then probed in either the absence or presence of either kethoxal (**A**) or CMCT (**B**). The RNA samples were reverse transcribed and the reactions then fractionated on 8% denaturing PAGE gels. The accessibility of either G (kethoxal and CMCT) or U (CMCT) was visualized by the presence of bands that downshift one nucleotide as compared to either a G or a U ladder produced using an untreated wild-type ribozyme with either dideoxy ATP (ddA) or dideoxy CTP (ddC) during the reverse transcription step. The positions of xylene cyanol dye (XC) and of both the G and the U nucleotides of the ribozymes are indicated on both gels.

In order to shed some light on exactly when the formation of the *t*WW GU base pair occurs, chemical probings using both kethoxal and 1-cyclohexyl-(2-morpholinoethyl)-carbodiimide metho-*p*-toluenesulfonate (CMCT) were performed. Kethoxal covalently modifies both the N1, the chemical group involved in the H-bond formation of the *t*WW GU base pair (see Figure 1), and the N2 groups of guanosine [30]. However, the kethoxal reaction is not informative for investigating the possibility of the formation of a *t*WH base pair prior to the cleavage step (i.e. one that involves only the O6 group of guanosine). CMCT reacts primarily with both the N3 and N1 groups of uridine and guanosine, respectively [30], thereby modifying two of the chemical groups that are involved either in the H-bonds between the U_23_ and the G_28_ of the *trans* Watson-Crick base pair, or in the H-bond involving the N3 group of U_23_ in the *trans* Watson-Crick/Hoogsteen base pair (Figure 1). In addition, these modifications also prevent single-stranded nucleotides from being reverse-transcribed, thus creating a stop that results in the appearance of a band one nucleotide before that of the modified guanosine or uridine. Inactive mutated ribozymes that halt the folding pathway at various steps (see Figure 1B and the Introduction), as well as a post-cleavage version, were incubated either in the absence or the presence of the chemical reagent prior to being reversed transcribed. The resulting reaction mixtures were then analyzed on denaturing PAGE gels. Instead of using *trans*-acting HDV sequences, *cis*-acting versions were probed so as to favour the structural homogeneity of each mutant. Moreover, the RNA samples were pre-incubated in the presence of MgCl_2_, a step which has been shown to be essential for some of the folding steps, to favour folding into an active conformation. Each *cis*-acting construct harboured a 3′-end extension which had no significant impact on the ribozyme’s structure according to previous probing experiments [31], but did permit the efficient binding of the antisense oligonucleotide used for the primer extension. Typical autoradiograms obtained are shown in Figure 4 for both the kethoxal and CMCT probings. In the absence of the chemical reagent no significant background was observed for all of the mutant ribozymes, with the exception of the cleaved-product ribozyme that always provided a more complex banding pattern (Figure 4A, B). In the presence of kethoxal, modification of most of the single-stranded guanines was observed while the double-stranded ones remained unaffected (e.g. stems I and IV), as expected. This indicates that the various mutated *cis*-acting ribozymes were properly folded. It is also evident that the region of both the pseudoknot 1.1 and the homopurine base pair (G_40–42_) seemed to vary in accessibility along the folding pathway, suggesting that these regions are highly dynamic as has already been reported [31]. The G_28_ residue appeared to be highly accessible to the kethoxal reaction in all six of the pre-cleavage mutants, but not in the cleaved-product construction (Figure 4A). Quantification of these results indicated a decrease in the intensity of the G_28_-band of at least 2.5-fold in the post-cleavage complex as compared to any of the pre-cleavage ones (i.e. varied from 2.5- to 5-fold depending on the construct; data not shown).

Unlike kethoxal, CMCT reacted to a lesser degree with the single-stranded guanines, in accordance with the literature (Figure 4B). However, a decrease in the accessibility of G_28_ in the post-cleavage *cis*-acting ribozyme was also observed, although to a lesser extent (2- to 3-fold) than in the kethoxal experiments. The CMCT probing of U_23_ was more challenging as the U_23_ probing signal was observed to be less intense than expected in all mutants (Figure 4B). This may reflect the role of this uridine in the stacking of stem I, the first step in the folding pathway of the HDV Rz. It may also be an indication of the presence of a weak *t*WH base pair that involves the N3H of the uridine (see Figure 1A inset) prior to the cleavage step. Quantification of the accessibility of the residue in the different mutants showed a decrease in the signal of 2- to 2.5-fold in the post-cleavage complex as compared to those of all of the pre-cleavage complexes, with the exception of the G_80_C-C_76_G mutant that yields a band of equivalent relative intensity. No signal was detected for U_23_A, as was expected, since the mutation replaces U_23_ by A_23_. Importantly, taken together, these results indicate that both the U_23_ and the G_28_ of an antigenomic HDV Rz version could form a *trans* Watson-Crick/Hoogsteen base pair before cleavage, and a *trans* Watson-Crick base pair only after the cleavage occurs.

In a recent publication, results from both crystal structure and MD simulation showed that the mutation of the catalytic cytosine (C_76_) could impair the formation of the *t*WW GU base pair, potentially leading to a misinterpretation of the results [21]. Since three of the mutants used in this study contain this type of mutation of C_76_ (i.e. the mutants G_80_C-C_76_G, ΔU_77_-C_76_G and C_76_G), this might explain why the *trans* Watson-Crick base pair formation appears to occur only after the cleavage step. In order to verify this hypothesis, the experiments were repeated using a *trans*-acting version of the first two mutants (G_80_C-C_76_G, ΔU_77_-C_76_G) in which the C_76_ was preserved. In addition, these ribozymes were pre-incubated in the presence of an uncleavable substrate in which the adenosine located in position −1 was replaced by a deoxyriboadenosine (SdA_−1_). In the case of the third mutant (C_76_G), the preservation of C_76_ involves working with the wild-type ribozyme in the presence of either the SdA_−1_ analogue, or with the 7 nt 3′-end product in order to confirm the formation of the post-cleavage complex. Both the kethoxal and CMCT probings yielded similar observations, namely that the accessibilities of both U_23_ and G_28_ were significantly reduced in the post-cleavage complex and that U_23_ was possibly involved in an interaction in the pre-cleavage step. This indicated that the *trans* Watson-Crick base pair formation likely results from the switching of a *trans* Watson-Crick/Hoogsteen base pair before the cleavage step to a *trans* Watson-Crick base pair after the cleavage, rather than being a requirement in order for the cleavage to take place (data not shown).

### Localization of Mg^2+^ Cations by Metal Ion-induced Cleavage

HDV Rz depends on the presence of divalent metal ions for both its proper folding and its catalysis to occur. Three distinct magnesium cations have been roughly located in the HDV Rz: one between stems I and III, another positioned near the GU Wobble base pair located at the bottom of stem I [11] and the third around the IV/II junction [32]. Furthermore, recent crystallographic data of a genomic ribozyme suggests that a unique Mg^2+^ ion interacts with both the *t*WW GU of loop III and the bottom of stem I [20]. That said, the localization of these metal ions along the entire HDV ribozyme’s folding pathway has yet to be reported. In order to address this issue, magnesium-induced cleavage probings were performed. This method is based on the fact that at higher pH, the water molecules surrounding a magnesium ion are more acidic than free ones [33]. The resulting hydroxide-surrounded magnesium ion can therefore act as potent nucleophile, removing protons from the 2′-OH of the ribose residues. As a consequence, the flexible RNA backbone surrounding the bound magnesium can be cleaved, via an in-line attack of the 5′-phosphate, by the resulting 2′-O^−^ of the ribose moieties. This technique has been successfully used for the detection of a coordinated Mg^2+^ located at the bottom of stem II in the antigenomic HDV Rz [32].

**Figure 5:**
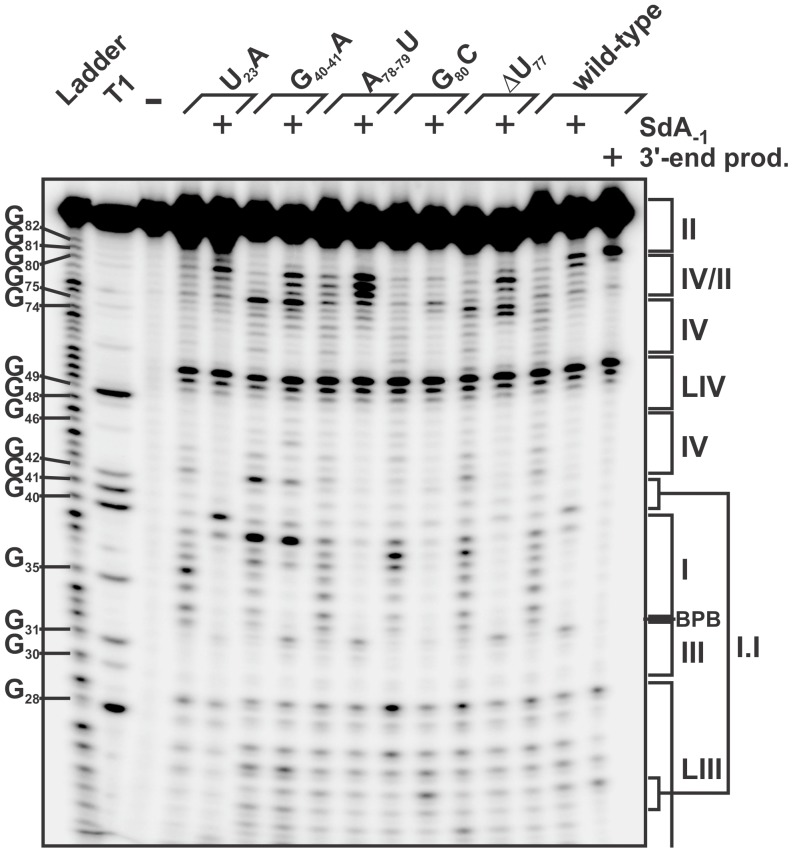
Global magnesium localization along the HDV Rz’s folding pathway studied by magnesium-induced cleavage. Different 5′-end-labeled *trans*-acting mutant ribozymes that halt the folding pathway at each known HDV Rz folding intermediate were folded either in the absence or the presence (+) of SdA-1 substrate or 3′-end product. The probings were then allowed to proceed for 48 h at room temperature in the presence of 50 mM Tris-HCL (pH 8.3) and 20 mM MgCl_2_. A control reaction without MgCl_2_ (−) was also performed. The resulting probings were analysed on 8% denaturing PAGE gels. The positions of bromophenol blue dye (BPB) and of the different regions of the Rz are indicated on the right of the gel. The lanes designated “Ladder” and “T1” represent an alkaline hydrolysis and a ribonuclease T1 (RNase T1) mapping of the wild-type version of the ribozyme, respectively. Representative guanosine residues are indicated on the left of the gel.

*Trans*-acting Rz versions of all of the mutants located along the folding pathway were used to perform the Mg^2+^ induced cleavage probings. Conversely to the chemical probings that were performed with *cis*-acting sequences that also required the substitution of the C_76_ by G_76_ in the cases of both the G_80_C and ΔU_77_ mutants in order to produced Rz lacking any cleavage activity, here the mutation of the catalytic cytosine was not required because the experiments were performed using *trans*-acting ribozyme with an uncleavable substrate. All mutants were 5′-end ^32^P-labeled, mixed or not with the uncleavable SdA_−1_ analog and then incubated in slightly basic buffer for 48 h in the presence of MgCl_2_. In order to obtain information about the post-cleavage product, the active ribozyme was also incubated in the presence of the 3′-end product. In all cases, the resulting samples were fractionated on denaturing PAGE gels. At first glance, the phosphodiester backbone remained intact in the absence of magnesium ion (Figure 5, lane -), suggesting that the hydrolysis banding patterns observed for all of the other reactions likely implied the specific binding of the metal ion. Overall, similar banding patterns were observed for all of the complexes. For example, stem I appeared to be protected to a greater extent in the presence of either the SdA_−1_ analog or the 3′-end product, in agreement with the fact that this region became double-stranded in all cases. Furthermore, the nucleotides of stem IV seem to be less flexible when stem I is formed, suggesting that the formation of this latter stem results in a more stable ribozyme structure [32]. However, a noticeable difference in terms of band number can be observed for the post-cleavage complex as compared to the pre-cleavage complexes. This is in accordance with an earlier report that a conformational switch controls HDV Rz catalysis and leads to a more compact post-cleavage structure [7]. Moreover, two main regions exhibit a relatively high accessibility in almost all of the reactions: the regions of loop III and junction IV/II that are primarily single-stranded prior to the formation of any specific tertiary interactions along the folding pathway and that are known to bind magnesium ions [11]. The sole exception is the G_80_C mutant in which the junction IV/II was less accessible than in the other mutants. This result could be explained by the necessity of conserving the G_80_ for the proper binding of the magnesium ion leading to the base pair-switch at the bottom of stem II. The third putative magnesium-binding region, namely the bottom of stem I, seems to be less flexible, although both U_39_ and G_40_ are sensitive to cleavage in a few of the reactions. Once again, the main difference in the banding patterns was observed with the post-cleavage complex. This difference is due to the total absence of any induced-cleavage surrounding this region, suggesting that this putative magnesium binding site either became constrained, or that this metal ion was ejected after the cleavage step (Figure 5, middle of the gel). Taken together, these results support the presence of magnesium ions in these three regions of the ribozyme.

Detailed analysis of the banding patterns of the residues forming both the junction IV/II and the loop III regions revealed several differences between the various mutants. Firstly, only U_77_ (which is involved in the trefoil-turn) and G_81_ (which is bulged out after the bp-switch) were accessible in the the post-cleavage complex’ junction IV/II, whereas all of the nucleotides in the various pre-cleavage complexes were accessible, although to different degrees (Figure 5, upper part of the gel). This result suggests that the magnesium ion located near junction IV/II is only stabilized in the post-cleavage complex, in agreement with previous reports [8], [32]. All known structural tertiary interactions, including the post-cleavage formation of the *t*WW GU base pair located within loop III, are likely required for the proper binding of this metal ion. In the case of loop III, although the band intensities are weaker than for those of junction IV/II, significant modifications in the banding patterns obtained along the folding pathway were observed. The intensities of the bands corresponding to nucleotides 23 to 27 were found to be faint, as compared to those of the other pre-cleavage products (Figure 5, bottom of the gel; nucleotides U_23_, C_24_, C_27_ and, to a less extent, both U_26_ and G_28_) with both the initial U_23_A Rz mutant and the post-cleavage complex. Considering that the magnesium ion has been demonstrated to be absolutely required for the docking of the substrate, a process which is prevented in the U_23_A Rz mutant, and that U_23_ has been shown to be in close proximity to the nucleotides located in the middle of the substrate strand (specifically nucleotide +4) by cross-linking experiments [4], it seems reasonable to suggest that a Mg^2+^-cation is bound in loop III at this initial step that follows the binding of the substrate to the ribozyme. More likely, interactions between the loop III residues and this specific Mg^2+^-cation are modified when the *t*WW GU base pair is formed in the post-cleavage product, and would in turn result in a modification of the banding pattern for this region. Alternatively, it may be possible, although less probable, that this Mg^2+^ cation is chased out and replaced by another that specifically interacts with the *t*WW GU base pair.

Finally, it was observed that the nucleotide C_29_ was accessible along the entire length of the folding pathway, suggesting that this residue is either highly flexible or is always bulged out. This nucleotide has been shown to be among the least conserved of loop III in SELEX experiments performed with the antigenomic HDV Rz [18], likely demonstrating that it is not involved in any specific interactions. In summary, the binding of metal ions implies a structural rearrangement of HDV Rz, especially for both the loop III and junction IV/II regions. This is particularly evident in the post-cleavage complex, and the positioning of the magnesium ions prior to cleavage seems to be more dynamic and less stringent than it is after, implying the putative formation of the *t*WW GU base pair.

### Structural Modeling of Both the Pre- and Post-cleavage Complexes

The three-dimensional representation of specific interactions among RNA molecules is a powerful tool in the interpretation of kinetic data. For this reason, 3D modeling was performed both before and after the cleavage step of the *cis*-acting antigenomic HDV Rz using MC-Sym [34]. This software uses cyclic building blocks extracted from crystallographic data to solve a constraint satisfaction problem based on the secondary structure. It has been successfully used in the 3D modeling of the various pre-cleavage intermediates along the HDV ribozyme’s folding pathway [31]. In order to access the conformational changes taking place during the cleavage reaction, a set of MC-Sym modelisations was designed based on the known interactions found in both the pre- and post-cleaved structures. In the case of the pre-cleaved structures, a *cis*-acting sequence that included four additional nucleotides located upstream of the cleavage site was used. Three scripts were written to model the structures present right before the cleavage reaction. These scripts include all of the features known to be required for the production of an active ribozyme, namely the substrate docking, the pseudoknot I.I, the A-minor motif, the GC base pair-switch and the trefoil-turn. The first script introduces the *t*WH GU base pair with the G_28_ in *anti* into the pre-cleavage structures, while the second and the third introduce the *t*WW GU base pair, with the G_28_ in *syn* and no GU base pair (negative control), respectively. In the case of the post-cleaved structures, a sequence identical to that used for the pre-cleaved structures was employed, except that it was shortened by four residues at the 5′-end (i.e. the sequence corresponding exactly to that of the cleaved *cis*-acting ribozyme). The same scripts as above, with the exception of the negative control, were used to model the structures present right after the cleavage reaction and included the same structural features as for the pre-cleaved structures. All of the manually scripted code lines required for the tertiary structure’s GU base pairs can be found in Supporting Information S1.

**Figure 6:**
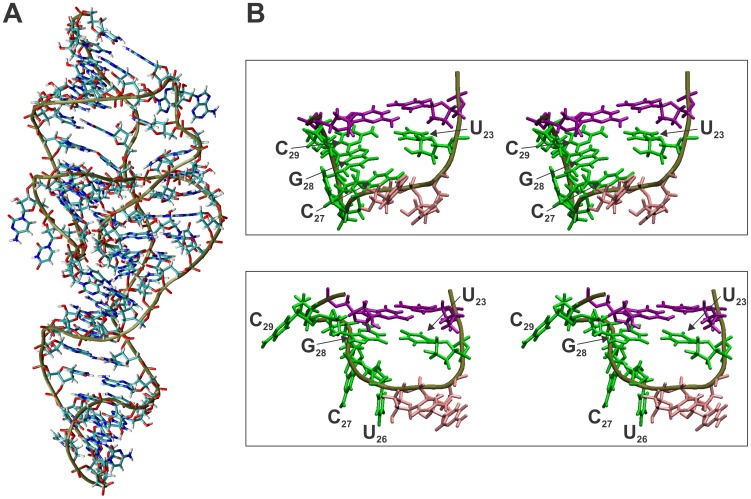
MC-sym structure depicting the formation of the *trans* Watson-Crick GU base pair after the cleavage step. (**A**) Representative structure of the post-cleavage HDV ribozyme. (**B**) Closer view of stereodiagrams of loop III both before (containing a *t*WH GU base pair, upper panel) and after (containing a *t*WW GU base pair, lower panel) the cleavage step. The colors are harmonized as in Figure 1.

Each of these five different scripts yielded a comparable number of structures, varying between 8 to 12, thus arguing that the tertiary structures based only on the nucleotides secondary and tertiary interactions cannot explain the post-cleavage stabilization, at least using the MC-Sym software. This result also demonstrates the relative flexibility of loop III, more specifically its ability to tolerate different structural conformations. After minimization, all of the structures obtained were visualized using the Visual Molecular Dynamics (VMD) software [35]. The most representative structures of both the pre and the post-cleavage families were selected based on both their stabilities and their average root-mean-square deviations (see Figure 6A). In general, the overall highly compact structures obtained were similar to those obtained previously by crystallography [6]. Briefly, all of the known tertiary interactions or structural rearrangements that take place in HDV Rz are visible, demonstrating that MC-Sym modeling has the ability to yield structures that satisfy the complex network of interactions of the HDV ribozyme.

A closer look at loop III revealed several interesting features in both the pre- (*t*WH GU base pair) and post- (*t*WW GU base pair) cleavage structures (Figure 6B). For example, C_29_ is bulged out in the post-cleavage structure and is therefore not involved in any specific interaction, in agreement with the results obtained in the Mg^2+^-induced cleavage (Figure 6B, lower panel). In the pre-cleavage structure, this nucleotide seems to be highly dynamic. Based on these results, C_29_ is not always stacked between G_28_ and G_30_ as depicted in figure 6B (upper panel), proving that this region is highly flexible and might be stabilized by the presence of a magnesium ion (data not shown). This stabilization could result in the definitive bulging-out of this C_29_, as occurs in the post-cleavage structure (Figure 6B, lower panel). The *t*WW GU base pair, as compared to the *t*WH GU base pair (Figure 6B, upper panel), appears to be stacked under stem III in the most compact structures and seems to trigger, in turn, the stacking of both U_26_ and C_27_ under itself. Most likely, these stacking interactions result in a more stable, structured loop III, a hypothesis which is confirmed by the hydrolysis patterns observed during the magnesium-induced cleavage experiments (i.e. a decrease in the degree of hydrolysis for all of these nucleotides in the post-cleavage structure).

## Discussion

The two nucleotides involved in the G_28_U_23_ base pair were shown to be perfectly conserved in all natural variants, and cannot be mutated individually without it being detrimental to the cleavage level. In fact, they are considered as being part of the *signature* of the catalytic core of HDV Rz of both the genomic and antigenomic polarities [17]. Although both x-ray diffraction and nuclear magnetic resonance experiments have provided high-resolution information on the tertiary structure of the HDV Rz [6], [7], [19], [20], including evidence of the formation of either a *t*WH or a *t*WW GU base pair in loop III, as yet no direct experimental result about the potential interaction between these bases (nor on the roles of the specific chemical groups within this base pair) has been obtained in solution. To our knowledge, this report represents the first time that these nucleotides were simultaneously mutated. Only a limited set of these mutants was found to be active, and even then the resulting active ribozymes exhibited significantly less cleavage activity than did the natural base pair (U_23_/G_28_) (Figure 2). The only double mutant exhibiting activity was the base combination C_23_/A_28_, which has the ability to form a *trans* Watson-Crick base pair isosteric to the *t*WW U_23_/G_28_ base pair present in the wild-type ribozyme version. All other enzymatically active mutants resulted from the single substitution of either U_23_ or G_28_. The resulting active nucleotide combinations all have the potential to form isosteric or near-isosteric base pairs in the *t*WW geometry as well as a *t*WH found in the pre-cleavage Rz crystal. However, the concept of isostery is not the sole parameter that is important to this base pair as some isosteric mutants (e.g. G_23_/U_28_ and A_23_/C_28_) have been demonstrated to be inactive. In fact, it has been already proposed that the structural flexibility of loop III in the genomic HDV Rz is gated by the closing of this *t*WW GU base pair. This closing allows not only a protonated C_75_ H^+^-induced conformational switch, but also creates an electrostatic environment that influences both the catalytic cytosine’s strength and the proper metal ion binding [36]. Consequently, the spatial locations of the functional groups involved in the positioning of the magnesium ion (O6 and N7 of G_28_ and O2 of U_23_) appear also important. This requirement can also explain the putative switch from the *t*WH base pair with the G_28_ in the *anti* configuration to the *t*WW base pair with the G_28_ in the *syn* configuration (see Figure 1A inset). A partial unfolding of loop III would be required in order to flip G_28_ from 180° and thus allow for the correct positioning of its Hoogsteen edge, which is involved in the binding of the magnesium ion, without disrupting the sole H-bond between the O6 of G_28_ and N3H of U_23_. Furthermore, the resulting *t*WW GU base pair produces a second H-bond between the N1H of G_28_ and the O4 of U_23_, thereby stabilizing the base pairing. Consequently, the concept of relative H-bond stability before (weaker) and after (stronger) the cleavage step seems to be an important parameter that could explain the extreme conservation of both of these nucleotides in nature. Moreover, it could explain why mutants allowing the formation of Watson-Crick base pairs prior to the cleavage step are not active. For example, the slightly active U_23_/A_28_ mutant can form 2 H-bonds by conventional *cis* Watson-Crick base pairing that should be difficult to break in order to form a *trans* Watson-Crick base pair as is found in the cleaved product.

The substitution of specific chemical groups provides physical evidence supporting the formation of a post-cleavage *t*WW GU base pair, or at least that of a traditional GU Wobble, and not a classical Watson-Crick, base pair (Figure 3). The removal of the NH_2_ group, which is linked to the C2 of guanine (i.e. it is involved in H-bond formation inside of a *cis* Watson-Crick GC bp), as occurs when an inosine residue is inserted in position 28, did not impair the cleavage activity. The absence of the N7 on the Hoogsteen edge of the 7-deazaguanine resulted in a relatively significant reduction in the cleavage activity, supporting the notion of the binding of the Mg^2+^ ion to the N7 chemical group of G_28_. This conclusion is also supported by the results of the magnesium-induced cleavage experiment in loop III. Together, the data strongly suggest the localization of a cation near loop III, more specifically near G_28_. Importantly, both the latter experiment and the chemical probings with both kethoxal and CMCT in the antigenomic version studied revealed that the *t*WW GU base pair is formed solely in the post-cleavage ribozyme’s catalytic core, and that possibly a *t*WH GU base pair forms in the pre-cleavage steps, although there is no experimental evidence in this study that confirms the presence of the *trans* Watson-Crick/Hoogsteen base pair before cleavage. Consequently, the results presented in this study undeniably point to the formation of the *t*WW GU base pair being a post-cleavage event.

The experiments performed in this study shed some light on the nature of the interaction that takes place within loop III, as well as on its timing with respect to the folding pathway. Since the *t*WW GU base pair was only detected in the post-cleavage complex, either it is formed after the chemical step (within the post-cleavage step) or during the transition complex. Since the interaction takes place so late along the folding pathway, it is reasonable to question its exact contribution to the molecular mechanism. As revealed by the MC-Sym modeling, the satisfaction of the distance constraint required for the formation of a *t*WW GU base pair leads to the formation of a more structured loop III (Figure 6). However, this appears to be more a consequence of, rather than a contribution to, the mechanism. It is tempting to speculate that the formation of two H-bonds in the *t*WW GU base pair, as well as the restructuration of loop III and the proper positioning of the magnesim ion, most likely stabilize the ground state of the post-cleavage complex and in turn favours the forward reaction. This hypothesis is supported by a recent crystal analysis which proposed that the genomic HDV Rz possesses an exit site for the 5′-end cleavage product for which no direct interaction with the ribozyme’s core has been reported to date [20]. This exit site is composed of five nucleotides, including G_25_ (G_28_ for the antigenomic Rz). The exit site points towards the PO_4_ group of the 5′-end cleavage product allowing for the fast release of the 5′-end product. This is in agreement with the absence of any reported ligation reaction for the HDV ribozyme. To our knowledge, this is the first time that an interaction that occurs after a ribozyme’s cleavage step may provide an additional driving force for the reaction to be unidirectional.

The formation of the *t*WW GU base pair in loop III constitutes one more step along the folding pathway of the HDV ribozyme, a folding pathway that has received significant attention over the years. This pathway includes 6 steps that occur prior to the cleavage event, from the formation of the substrate-ribozyme complex to the formation of the trefoil-turn within junction IV/II (see Figure 1). Most likely, simultaneously with the cleavage step, the 5′-end product is released and the *t*WW GU base pair is formed. The formation of this *t*WW base pair may also help release the 5′-end product. Lastly, the 3′-end product, which has been shown to remain bound to the ribozyme under certain conditions, is eventually released. This folding pathway appears to be linear; however, kinetic traps that can be reintegrated into the productive pathway have also been reported [4]. Regardless, the formation of the *t*WW GU base pair most likely occurs after the cleavage step, and may serve to “drive” to the end-point of the catalysis.

**Figure 7:**
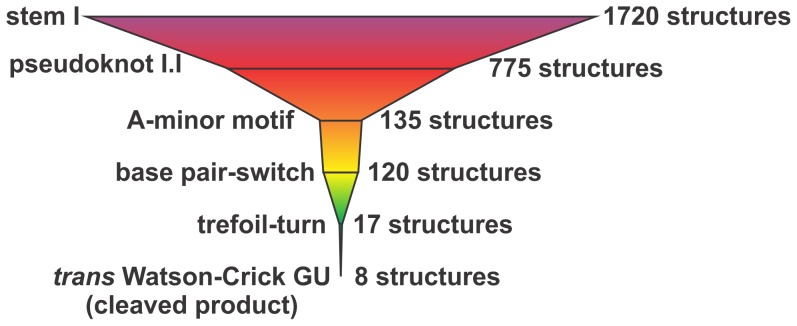
Representation of the number of putative structures as a function of constraints used in the modeling performed with MC-Sym.

An RNA molecule possesses a hierarchical structure in which the primary nucleotide sequence determines the secondary structure, which, in turn, determines the tertiary folding in a process that only minimally alters the secondary structure. More specifically, the molecule folds sequentially from 5′ to 3′. The folding intermediates tend to become increasingly stable during this process, and therefore follow a funnel process. However, the number of possible distinct structures that can be retrieved along this process is unknown. In order to provide an idea of exactly how the different tertiary interactions contribute to reducing the number of distinct structures, MC-Sym modeling experiments were performed for each intermediate using a calculation time of 240 h so as to permit MC-Sym to find all of the potential structures and not only the best ones (i.e. to tend towards the saturation of the number of possible structures). This process yielded 1750 structures for the initial step of the formation of the substrate-ribozyme complex (i.e. through stem I formation), and only 8 for the post-cleavage complex that included the *t*WW GU base pair (Figure 7). This experiment provides an enrichment of over 200-fold, which is actually underestimated as a constant increase in the number of structures with time was observed for the initial step (i.e. no tendency towards saturation). Moreover, the free ribozyme (i.e. before the formation of stem I) is known to be relatively flexible; consequently, it can adopt a variety of distinct structures. More importantly, the number of structures was reduced at each successive step, as expected. It should be noted that this remains a simplistic manner in which to estimate the number of structures, and it is important to consider that there are no results supporting the existence of all of these distinct species. Furthermore, the irrelevant structures, including the ones with very low probabilities of being retrieved, were not removed from the samplings. It is also possible that some tertiary structures may occur in a different order depending on the folding pathway followed, a fact not taken into consideration here.

## Materials and Methods

### Ribozyme DNA Templates

The DNA templates corresponding to the mutated ribozymes were produced using two overlapping DNA oligonucleotides (Forward oligonucleotide: 5′-TAATACGACTCACTATAGGGTCCACC(N)CCTC(N)CGGTCCGACCTGGGCATGCGGCTTCGC-3′ and Reverse oligonucleotide: 5′-GGGTCCCTTAGCCATGCGAAGCCGCATGCCCAGGTCGGACCG-3′, in which the underlined nucleotides represent the T7 promoter sequence that was included so as to permit RNA transcription and N represents any of the four possible nucleotides). The same strategy was used for the production of *cis*-acting ribozymes, except that the Forward oligonucleotide contained an extra sequence (either 5′-GGGCTAAGGGTCGGCA-3′ or 5′-GGGTCGGCA-3′ (cleaved product) immediately following the T7 promoter sequence. The Reverse oligonucleotide also contained an extra sequence (5′-GTTTGTTTGTTTGTTGAGG-3′) located in the 3′-end region of the ribozyme to permit the annealing of a DNA oligonucleotide during reverse transcription. The filling reactions were performed in a final volume of 100 µL containing 20 mM Tris-HCl (pH 8.8), 10 mM KCl, 10 mM (NH_4_)_2_SO_4_, 2 mM MgSO_4_, 0.1% Triton X-100, 200 µM of each dNTP, 1 µM of each DNA oligonucleotide and 5 units of *Pwo* DNA polymerase (Roche Diagnostics). The reaction products were ethanol precipitated, washed and the resulting DNA pellets dissolved in 56 µL of deionized water.

### RNA Synthesis

RNA transcriptions were performed as described previously [31]. Briefly, the dissolved DNA pellets (56 µL) were used in 100 µL transcription reactions containing 80 mM HEPES-KOH (pH 7.5), 24 mM MgCl_2_, 2 mM spermidine, 40 mM DTT, 5 mM of each NTP, 0.01 unit of pyrophosphatase (Roche Diagnostics), 20 units of RNaseOUT RNase inhibitor (Invitrogen) and 10 µg of purified T7 RNA polymerase. The reactions were incubated for 3 h at 37°C, and were then treated with 4 units of RQ1 DNase (Promega) prior to being phenol-chloroform extracted. The RNA was then ethanol-precipitated, washed and finally dissolved in 40 µL of deionized water. Loading buffer (40 µL; 97.5% formamide, 0.05% bromophenol blue, 0.05% xylene cyanol, 10 mM EDTA) was then added and the samples fractionated on 8% denaturing (8 M urea) polyacrylamide gels (PAGE, 19∶1 ratio of acrylamide to bisacrylamide) using 45 mM Tris-borate (pH 7.5) and 1 mM EDTA solution as running buffer. The RNA was visualized by UV shadowing, the gel slices corresponding to the desired bands were cut out and the RNA eluted overnight in 500 mM ammonium acetate, 1 mM EDTA and 0.1% SDS solution. After ethanol precipitation, the RNA transcripts were dissolved in deionized water and quantified by spectrometry at 260 nm. Deprotected RNA Substrate (5′-CUAAGGGUCGG-3′), SdA_−1_ analog (5′-CUAdAGGGUCGG-3′) and 3′-end cleavage product (5′-GGGUCGG-3′) were purchased from IDT. RNA-DNA mixed ribozymes including modified residues were purchased from Dharmacon, deprotected and purified on PAGE gels as described above.

### Radioactive Labeling of Both RNA and DNA Species

Purified ribozymes (50 pmol) were dephosphorylated using 5 units of Antarctic phosphatase as prescribed by the manufacturer (New England BioLabs), followed by heat inactivation of the enzyme for 8 min at 65°C. The dephosphorylated RNA (5 pmol) was 5′-end labeled by incubating it at 37°C for 1 h in a final volume of 10 µL containing 3.2 pmol of [γ-^32^P]-ATP (6000 Ci/mmol, New England Nuclear), 50 mM Tris-HCl (pH 7.5), 10 mM MgCl_2_, 50 mM KCl and 3 units T4 polynucleotide kinase (USB). The reactions were stopped by adding 10 µL of loading buffer (97.5% formamide, 0.05% bromophenol blue, 0.05% xylene cyanol, 10 mM EDTA), and the RNA purified by denaturing PAGE as described above. The 5′-end labeling of either the RNA substrates or the DNA oligonucleotides used in the reverse transcription reactions was performed using 5 pmol as described above, and the products purified by 20% denaturing PAGE.

### End-point Cleavage Assays

End-point cleavage assays were performed by preparing 18 µL reactions containing trace amounts (≤1 nM, 50,000 CPM) of 5′-end labeled substrate and 2 pmol of the desired ribozyme (100 nM final concentration) and then heating at 65°C for 2 min, followed by 2 min on ice and 5 min at 37°C. Then, 2 µL of a solution containing 500 mM Tris-HCl (pH 7.5) and 100 mM MgCl_2_ were added and the entire reaction was incubated at 37°C for 2 h. The reactions were stopped by the addition of 20 µL of loading buffer, fractionated on 20% denaturing PAGE gels which were then exposed to PhosphorImager screens and scanned using a Typhoon apparatus (GE Healthcare). The activity of each ribozyme was determined using the ImageQuant (Molecular Dynamics) software, and is expressed as the percentage of cleaved product counts over total counts. Each end-point was calculated from the results of at least 2 independent experiments.

### Kinetic Analyses

Kinetic analyses were performed under single-turnover conditions as described previously. Briefly, trace amounts of 5′-end labeled substrate (≤1 nM, 50,000 CPM) were cleaved by various ribozyme concentrations (6.25–1600 nM). The fractions cleaved were determined as described above and the rate of cleavage (k_obs_) was obtained by fitting the data to the equation A_t_ = A_∞_ (1-e^−kt^) where A_t_ is the percentage of cleavage at time t, A_∞_ is the maximum percent cleavage (or the end-point cleavage) and k is the rate constant (k_obs_). Each rate constant was calculated from at least 2 independent experiments. The values of k_obs_ obtained were then plotted as a function of ribozyme concentration to determine the other kinetic constants (k_2_, K_M’_, and k_2_/K_M’_). The magnesium dependency for each ribozyme was studied by incubating the reaction mixtures with various MgCl_2_ concentrations (0.5–64 mM) in the presence of an excess of ribozyme (500 nM) over substrate (≤1 nM, 50,000 CPM). The concentrations of magnesium at the half-maximal velocity (K_Mg_) were also determined.

### Chemical Probings

*Cis*-acting ribozyme (5 pmol) dissolved in water (18 µL) was heated at 65°C for 2 min, put on ice for 2 min and then incubated at 37°C for 5 min. A solution (2 µL) containing either 500 mM HEPES-KOH (pH 7.5), 100 mM MgCl_2_ and 100 mM NaCl for the kethoxal reaction, or 200 mM potassium borate (pH 8.0), 100 mM MgCl_2_ and 100 mM NaCl for the CMCT reaction, was then added and the reaction incubated at 37°C for 15 min. The chemical probing were initiated by adding 0.5 µL of either kethoxal (20 mg/mL in 20% ethanol; Aldrich) or 1-cyclohexyl-3-(2-morpholinomethyl) carbodiimide metho-*p*-toluenesulfonate (CMCT) (84 mg/mL in water; MP Biomedicals) and incubating the reactions at 37°C for 5 min. Negative controls were performed by adding 0.5 µL of either 20% ethanol (kethoxal) or water (CMCT) in place of the chemical agent. The reactions were quenched by the addition of 20 µL of 50 mM potassium borate (pH 7.0), and the RNA ethanol-precipitated in the presence of 10 µg of glycogen (Roche Diagnostics). The resulting precipitates were ethanol-washed and dissolved in 12 µL of 50 mM potassium borate (pH 7.0) containing 1 pmol of 5′-end radiolabeled DNA oligonucleotide (5′-GTTTGTTTGTTTGTTGAGGG-3′) (50,000 CPM). The samples were then heated at 65°C for 5 min, cooled at 37°C for 5 min and finally incubated at 4°C for 1 min. At this point, 4 µL of 5X First-Strand Buffer (Invitrogen), 1 µL of 100 mM DTT, 1 µL of 10 mM dNTP and 2 µL of DMSO were added and the reactions preincubated at 56°C for 1 min prior to adding 100 units of SuperScript III (Invitrogen) and incubating for another 20 min at 56°C. In the case of the ladder, untreated RNA and an additional 1 µL of either 10 mM ddCTP or 10 mM ddATP were used in the reaction. The reactions were stopped by adding 1 µL of 4 N NaOH, and were then heated at 95°C for 5 min so as to hydrolyze the RNA. The newly synthesized cDNA was then ethanol-precipitated, dissolved in 10 µL of loading buffer and fractionated on 8% denaturing PAGE gels. The gels were exposed to PhosphorImager screens, scanned using a Typhoon apparatus (GE Healthcare) and the band intensities quantified using the ImageQuant (Molecular Dynamics) software. The background from the reverse transcription reaction was subtracted using the result obtained with either the ethanol- (kethoxal) or the water- (CMCT) treated RNA. Both nucleotides U_23_ and G_28_ were quantified and normalized against the intensities of nucleotides U_51_ and G_49_ from loop IV for which the signals were constant regardless of the experimental conditions. Each chemical probing result presented was calculated from at least 2 independent experiments.

### Magnesium-induced Cleavage

Reactions (18 µL) containing trace amounts (≤1 nM, 50,000 CPM) of 5′-end labeled ribozyme either with or without 20 pmol of either uncleavable SdA_−1_ analog or 3′-end cleavage product (1 µM final concentration) were heated at 65°C for 2 min, put on ice for 2 min and then incubated at room temperature for 5 min. A solution (2 µL) containing 500 mM Tris-HCl (pH 8.5) and 200 mM MgCl_2_ was then added and the reactions incubated at room temperature for 48 h. A negative control (without MgCl_2_) was performed by adding 2 µL of 500 mM Tris-HCl (pH 8.5). Following the reaction, the RNA was ethanol precipitated in the presence of 10 µg of glycogen, and the resulting pellet then dissolved in 10 µL of loading buffer. For alkaline hydrolysis, 50,000 CPM of 5′-end labeled ribozyme (<1 nM) were dissolved in 5 µL of water, 1 µL of 2 N NaOH was added and the reaction incubated for 1 min at room temperature prior to being quenched by the addition of 3 µL of 1 M Tris-HCl (pH 7.5). The RNA was then ethanol-precipitated in the presence of 10 µg of glycogen and dissolved in 10 µL of loading buffer. An RNase T1 ladder was prepared using 50,000 CPM of 5′-end labeled ribozyme (<1 nM) dissolved in 10 µL of buffer containing 20 mM Tris-HCl (pH 7.5), 10 mM MgCl_2_ and 100 mM LiCl. The mixture was incubated for 1 min at room temperature in the presence of 0.6 unit of RNase T1 (Roche Diagnostic), and the reaction then quenched by the addition of 20 µL of loading buffer. The samples were fractionated on 8% denaturing PAGE gels which were then exposed to PhosphorImager screens.

### MC-Sym

All of the information required to generate, edit and optimize the scripts used in the MC-Sym program can be found on the MC-Pipeline webpage (http://www.major.iric.ca/MC-Pipeline). The three basic templates used to fold HDV ribozyme (one template for the secondary structure, one for the secondary structure including the pseudoknot I.I and one for the secondary structure including both the pseudoknot I.I and the GC bp-switch) have previously been published [31]. In all of these templates the order used to build the tertiary structures started with stem I, then stem III, loop III and the pseudoknot I.I (if present), followed by the bottom of stem II, junction IV/II, the top of stem IV, junction I/IV (when the pseudoknot I.I is not present), stem II, junction I/II, stem IV and finally the 5′-end of the ribozyme. All of the manually scripted code lines required for the tertiary structure of the GU base pairs can be found in the Supporting Information S1. At each position, 25% of the cyclic building blocks were tried, the backtrack was 25% of the structure and the method was probabilistic. The maximum number of structures was fixed at 10,000, the computation time to 240 h and the minimal difference between two structures to 1 Å. The energies of the structures were minimized until the root mean square of the gradient (GRMS) was <0.1 kcal/mol/A directly on the web server.

## Supporting Information

Supporting Information S1 **Manual editing of MC-Sym scripts in order to introduce specific GU base pairs into HDV ribozyme.**
(DOCX)
