# Supplementary material for: Characterization of the Trans Watson-Crick GU Base Pair Located in the Catalytic Core of the Antigenomic HDV Ribozyme
Source: PLoS One. 2012 Jun 29;7(6):e40309. doi: 10.1371/journal.pone.0040309 (PMC3386971; doi:10.1371/journal.pone.0040309)
Supplement: Supporting Information S1 — Manual editing of MC-Sym scripts in order to introduce specific GU base pairs into HDV ribozyme. (DOCX) [file pone.0040309.s001.docx]

**Supporting Information S1. Manual editing of MC-Sym scripts to introduce specific GU base pairs in HDV ribozyme.**

//========== Sequence ==========

sequence( r A1 CUAAGGGUCGGCAGGGUCCACCUCCUCGCGGUCCGACCUGGGCAUGCGGCUUCGCAUGGCUAAGGGACCC )

// ....(((((((..[[[[[[(((.......))))))))))..((((((((..)))))))).....]]]]]]

// CCCCCCC aaaaaaAAAAAAAAAAAAACCCCCCC BBBBBBBBBBBBBBBBBB aaaaaa

// | | | | | | | | | | | | | |

// 5 10 15 20 25 30 35 40 45 50 55 60 65 70

***trans* Watson-Crick/Hoogsteen U_23_G_28_ base pair with G_28_ in *anti* conformation**

//========== Relations ==========

relation(

//this constraint will define the trans watson hoogsteen U23G28

A23 A28 { pairing && 95} 10 )

// ========= Ribose / Restraints =========

//this restraint will define the anti conformation for the G28

ribose_rst ( structure[A28]

method = ccm,

pucker = C3p_endo,

glycosyl = anti,

threshold = 2.0 )

***trans* Watson-Crick U_23_G_28_ base pair with G_28_ in *syn* conformation**

//========== Relations ==========

relation(

//this constraint will define the trans watson watson U23G28

A23 A28 { pairing && XXVII} 10 )

// ========= Ribose / Restraints =========

// this restraint will define the syn conformation for the G28

ribose_rst ( structure[A28]

method = ccm,

pucker = C3p_endo,

glycosyl = syn,

threshold = 2.0 )
